# Supply chain movement risk in the sneaker industry: an empirical study

**DOI:** 10.1007/s11135-021-01166-y

**Published:** 2021-06-10

**Authors:** Che-Wei Chang

**Affiliations:** Department of Recreational and Graduate Institute of Recreational Sport Management, National Taiwan University of Sport, No. 16, Sec. 1, Shuang-Shih Road, Taichung, 404, Taiwan, Republic of China

**Keywords:** Supply chain management, Focus group, Entropy, Grey situation decision-making algorithm

## Abstract

In light of the COVID-19 pandemic and the Sino–US trade war, this study proposes a grey sharing decision-making evaluation model for production base movement and the sustainable operation of enterprises in the footwear industry. First, a focus group technique was employed; personnel from the footwear industry, corresponding government agencies, and the academic community were invited to jointly identify the most important criteria when considering a production base movement. The group listed seven criteria: labor cost, materials, exchange rate fluctuation, tariff, supply chain, transfer cost, and the government. The grey situation decision-making algorithm based on group knowledge and entropy were used to identify the most suitable country for production base movement.

## Introduction

The footwear industry is labor-intensive, and is affected by various factors such as land resources, labor costs, material supply, environmental protection, and sales markets. Consequently, in pursuit of profit maximization, major consumer markets, footwear manufacturers, wholesalers, and retailers worldwide are shifting their focus to lower-cost countries, leading to constant movement of global shoemaking bases (Kong Cheong Shoes Group 2014). For instance, European countries such as Italy, Spain, and Portugal began to move their shoemaking bases to countries or regions with lower costs, such as Japan, Taiwan, South Korea, and Hong Kong in the 1960s, and thereafter to China in the late 1980s. Since 1996, China’s footwear industry has been growing at a rate of 10–20% annually, and the country has become the world's leading footwear producer and exporter (Leo 2020). According to Wu (2018a) and the World Footwear Yearbook (2017) there were 30,000–40,000 shoemaking enterprises worldwide in 2016, employing nearly 10 million individuals and producing 23 billion pairs of shoes.

Taiwan's footwear industry has gone through five phases: (1) Early phase (1949–1960): Under the business strategy adopted during this period, the store and factory were located in the same place, with the main service being shoe customization. Each shoemaker could therefore make only one or two pairs of shoes per day (Zhou and Li 2004). (2) Burgeoning phase (1961–1969): Taiwanese footwear factories introduced Japanese shoemaking techniques. They began to apply the mass production model as well as the business strategy of separating production and sales. At the time, there were more than 30 export-oriented footwear factories in Taiwan, the annual average exporting 20 million pairs of shoes and constituting a major export industry in the country. (3) Maturity phase (1970–1988): Earlier in the maturity period, favorable factors such as low wages, abundant labor, and high quality attracted a large number of orders from footwear manufacturers outside Taiwan. The number of footwear manufacturers in the country surged to more than 1400, with the volume of footwear production growing annually by 50%. In 1987, exports reached a peak of 1.8 billion pairs, making Taiwan the world's second largest footwear exporter. However, since 1987, the New Taiwanese dollar appreciated sharply by 45%, wages rose, and the profitability ratio began to shrink. Manufacturers began to consider moving their production bases (Li 2017). (4) The industrial structure adjustment phase (1988–1989): This period witnessed high wages, rising labor consciousness, and labor shortages. Moreover, there was significant appreciation of the New Taiwanese dollar against the U.S. dollar, with US$1 being equivalent to NT$39.85 in 1985 and NT$26.42 in 1989. With this sharp appreciation of nearly 50%, profits and total output plummeted. During this period, manufacturers gradually moved their shoemaking bases to countries or regions with land and labor cost advantages such as Thailand, Vietnam, Hong Kong, and China (Xie 2017). (5) The International Division of Labor Phase (1990–present): From a globalization context, the number of shoemaking bases located in Taiwan has reduced drastically, whereas overseas investments have surged, forming a new pattern of international division of labor, whereby orders are received in Taiwan and shoes are produced outside Taiwan. Nevertheless, operational activities and research and development (R&D) centers have been retained to develop and design new shoe materials and models, and produce high value-added and differentiated products. Taiwan still holds the leading position in the global footwear industry (Chen 2015).

In each period, even though the footwear industry faced significant uncertainties such as environmental changes, local governments, and competition from international brands, Taiwanese enterprises gradually developed large-scale manufacturing capacity and manufacturing management capability, to balance quality and efficiency. To facilitate sustainable enterprise operation and development, it is desirable for production and supply chains to move to a cheaper country approximately every two decades. Recently, due to the COVID-19 pandemic and the Sino–US trade war, the exchange rate between the NT$ and US$ has changed dramatically, and many Taiwanese companies have begun to evaluate the feasibility of transferring production lines to countries with lower production costs. Therefore, this study proposes a grey sharing decision-making evaluation model (GSDEM) to analyze the movement of the production bases of original equipment manufacturers (OEMs), for the sustainable operation and development of footwear enterprises. For this purpose, first, the focus group method was used; personnel from the footwear industry, government, and academic community were invited to jointly identify the criteria for evaluating production base movement. Thereafter, the grey situation decision-making (GSDM) algorithm and entropy were used to construct the model. The results of this study can serve as a useful reference for the sustainable operation of shoemaking OEMs.

## Literature review

**Fig. 1:**
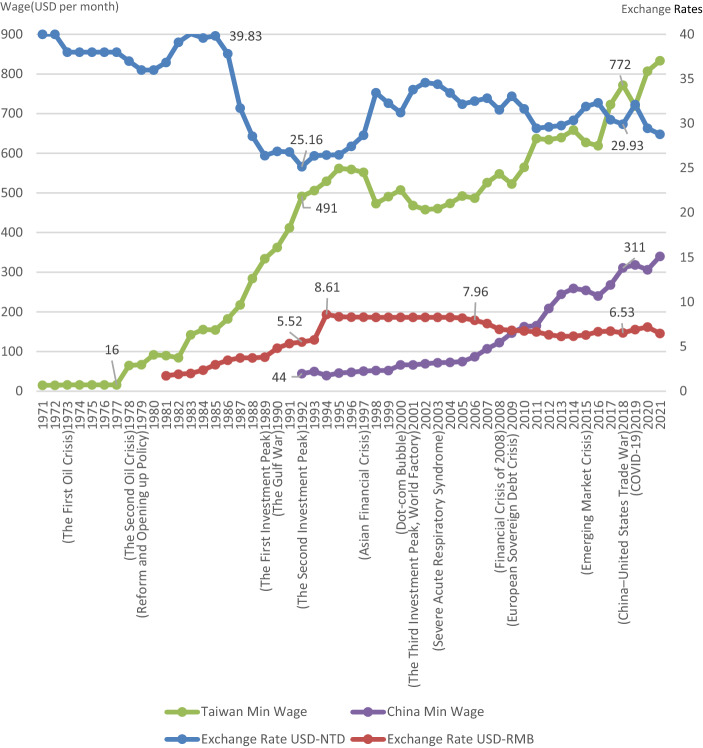
Fluctuations in wages and exchange rates in China and Taiwan since the 1970s and their effect on the supply chain movement risk in the sneaker industry

The shoe supply chain includes the sourcing of raw materials, transforming them into semi-finished goods (shoe materials, shoemaking, finished products), and branding (distributing goods to final users) (Marconi et al. 2017). Schwartz (2018) presented the sneaker supply chain, which starts with design and ends with the manufactured shoe, over an 18-month period. The risks faced by suppliers in the supply chain include increasing labor costs, national tax affairs policy, tariff, and the exchange rate. Figure 1 shows the fluctuations in the wages and exchange rates in Taiwan and China since the 1970s, representing the numerous supply chain movement risks in relation to the sneaker industry. For example, Taiwan’s exchange rate rose from 39.83 NT$: 1 US$ in 1985 to 25.16 NT$: 1 US$ in 1992, when China witnessed its second investment peak period since the country’s reform and opening up. This increase was as high as 58%. The minimum salary increased from US$16 per month in 1977 to US$491 per month in 1992, an increase of about 30 times. With both wages and exchange rates rising, it became increasingly challenging for the highly labor-intensive shoe industry to continue production in Taiwan. At this time, the supply chain of the shoe industry began to shift to China. During this period, the Taiwanese investment in China was characterized by a business model in which Taiwanese businessmen would take orders in Taiwan, conduct processing and manufacturing in China, and export the products (Wikipedia 2020). From 1994 to 2005, China adopted a stable policy of low exchange rates and a stable minimum wage. After the Asian financial crisis and the dot-com bubble, the average interest rate remained at 8.30 RMB: 1 US$, while the minimum wage ranged from US$39 per month in 1994 to US$75 per month in 2005. The stable exchange rate and low wages attracted foreign investment to China, heralding the golden age for the development of China's manufacturing industry (Wei 2005). In 2002, China produced products to become "the world's number one manufacturer," including within the shoe industry (Tung 2003). In 2013, 10 labor-intensive industries—textiles, clothing, shoes, sporting goods, scooters, toys, hygiene, lighting, heating, and furniture—achieved the highest exports, accounting for 39.3% of the world’s production volume of these goods. Mainland China has become the world's manufacturing plant, with complete supply chains in many industries (Wang 2021). Since the 2008 global financial crisis, which led to inflation, the average wage in China has increased by 8.2% per year (National Bureau of Statistics 2019). Xu (2019) reported that during the Sino–US trade war in 2018, the United States increased the number of shoes imported from China. However, the 10% tariff and the COVID-19 pandemic have caused the supply chain of the shoe industry to move from China to other countries.

Zhou and Li (2004) conducted a strengths, weaknesses, opportunities, and threats (SWOT) analysis of the competitiveness of the Pou Chen Group and found that the company became the world’s largest international shoe OEM due to its R&D advantages. Mamic (2005) conducted interviews with the managers of 22 multinational companies and 74 suppliers, and studied the selection of global supply chain vendors in the sports footwear, apparel, and retail sectors. The author found that the evaluation criteria included geographical location, product variety/quality, company size, and ability to adhere to a code of conduct. Camuffo et al. (2008) investigated the Italian footwear manufacturer Geox, and found that the original Geox breathes^®^ patented system, which allows for ventilation in waterproof rubber soles, made it difficult for competitors to launch counterfeit products through reverse engineering. In less than a decade, Geox has become one of the largest footwear manufacturers in the world. Sellitto et al. (2015) proposed a two-dimensional supply chain operational performance (SCOR) model that had SCOR processes (source, make, deliver, and return) and performance standards adapted from the original SCOR (cost, quality, delivery, and flexibility). This model had a 4 × 4 matrix structure, and an analytical hierarchy process to calculate the importance of each criterion, to determine the supply chain performance of the Brazilian shoe industry. Wu (2018b) employed the strategy management framework proposed by Lin and Pao (2004), McKinsey's 7S model (McDonald 2014), and Michael Porter's Five Forces Model (1980) to analyze a company's internal and external advantages and overall environmental advantages. The study also used the political, economic, sociological, technological, legal, and environmental (PESTLE) model (Puhakka and Sipola 2011; Colicchia et al. 2019) to evaluate the strengths and weaknesses of the overall environment, providing companies with strategies for efficient integration of the supply chain as well as business and competition strategies for creating added value for products. Table 1 compares the earlier studies, to facilitate an analysis of the barriers to implementing global supply chain management.

**Table 1:** Recent literature on sneaker industry supply chain

Author and year	Research objective	Methodology	Evaluation criteria	Findings
Zhou and Li (2004)	International shoemaking industry	SWOT	OEM and R&D	Globalization and vertical integration reducing shoe manufacturing costs
Mamic (2005)	Global supply chain vendors in the sports footwear, apparel, and retail sector	Interviews	Geographical location, product variety/quality, company size, ability to adhere to a code of conduct	Global supply chain systems applying to the sports footwear, apparel, and retail sectors
Camuffo et al. (2008)	Italian footwear manufacturer Geox	Case study	Strategic innovation	The related notions of complementarity and performance landscape were applied to product innovation, to study strategic positioning in the footwear industry
Chen et al. (2007)	Suppliers, manufacturers, distributors, and retailers in a multi-echelon supply chain	Data envelopment analysis	Information sharing scenarios	Information sharing can enhance the performance of supply chains and improve service levels, fulfillment rates, and order cycle time
Liang (2012)	Supply chains under an uncertain environment	Possibilistic linear programming method	Integrated manufacturing/distribution planning decision problems	Effectively improve manufacturer and distributor relationships in a supply chain
Sellitto et al. (2015)	Supply chain operational performance	Two-dimensional SCOR model	Source, make, deliver, return, cost, quality, delivery, and flexibility	A supply chain operations reference model to inform the global performance of the Brazilian footwear industry supply chain
Marconi et al. (2017)	Shoe supply chain	Integrated definition method	Labor cost, the national tax affairs policy, and the exchange rate	Extends the classical concept of supply chain traceability, to communicate to each actor and consumer the exact origin of each raw material, semi-finished part or final product

Chen et al. (2007) utilized data envelopment analysis (DEA) to evaluate the information quality of suppliers, manufacturers, distributors, and retailers in a multi-echelon supply chain on chain performance. Liang (2012) proposed a probabilistic linear programming (PLP) method to solve integrated manufacturing/distribution planning decision problems in an uncertain environment. The PLP method can effectively improve manufacturer and distributor relationships in supply chains in an uncertain environment. Modgil and Sonwaney (2019) applied Zolfani et al.’s (2013) hybrid approach of a multi-criteria decision-making model, step-wise weight assessment ratio analysis (SWARA) and weighted aggregated sum product assessment (WASPAS) methodology to prioritize the application of blockchain in different industries.

Deng (1989) proposed the grey theory, in which grey relational analysis (GRA) and GSDM could be used with different research methods to develop multi-attribute decision-making problems. GRA can be combined with entropy to find the best decision-making solution and consequently, improve the business performance of enterprises (Sun 2014; Wang et al. 2015; Jatav and Chaturvedi 2016; Jiang et al. 2017; Soni et al. 2019). Su (2002) used entropy to develop indicators of diversification and explored patterns of diversification in Taiwanese business groups. The study concluded that when the core industries of a Taiwanese enterprise grew slowly or the investment in the R&D of core industries was high, the enterprise tended to carry out non-related diversification. Nolan (2020) presented entropy and equivocation to objectively assess the relative and absolute information potential for cultural resource management practitioners, to more explicitly justify their recommendations for expenditure on public and private development funds. Ho and Tsai (2018) combined the GRA and the BCG matrix to develop a model for the sustainable operation and development of enterprises, and evaluated the competitiveness of 13 public sector organizations belonging to the Taiwan Sugar Corporation after diversification. GSDM can be used together with GRA (Kung and Wen 2007), fuzzy theory (Congjun et al. 2007), and prospect theory (Zhang et al. 2014) to examine multi-attribute decision-making problems in different situations in order to satisfy the demands of decision makers. Lin et al. (2018) used the modified Delphi method and analytic network process to construct a two-stage multi-criteria decision-making model to evaluate and select suppliers during the procurement of high-precision and high-priced tools in the aerospace industry.

## Grey sharing decision-making evaluation model

The GSDEM algorithm provides an effective means of addressing one event that involves multiple decisions. GSDEM is a very useful optimization model that follows an uncomplicated principle, features simplicity of use, and allows for handy calculations and reliable results (Deng 2000; Chang and William 2006; Lin et al. 2006; Tai et al. 2011; National Bureau of Statistics of the PRC 2020; Liang 2017). The GSDEM and entropy methods are defined as follows:

### Definition 1

Let *x*_*i*_, *i* = 1, 2, …, *n* be the measurable production base movement factor and *y*_*j*_, *j* = 1, 2, …, *m* be the participation in the evaluation needs of decision makers. Then, *x*_*i*_ and *y*_*j*_ are referred to as combined evaluation events, and *E*_*ij*_ refers to a decisive situation and is given by the following:

1 $${\varvec{E}}_{{{\varvec{ij}}}} = \left( {{\varvec{x}}_{{\varvec{i}}} ,\user2{ }{ }{\varvec{y}}_{{\varvec{j}}} } \right),$$

The production base movement evaluation of the effectiveness of a measurable factor is the target. Each evaluation factor has only one target.

### Definition 2

If ${{\varvec{E}}}_{{\varvec{i}}{\varvec{j}}}=\left({{\varvec{x}}}_{{\varvec{i}}},\boldsymbol{ }{ \, {\varvec{y}}}_{{\varvec{j}}}\right)$ is a production base movement scenario, then the effectiveness of *x*_*i*_ and *y*_*j*_ can be written as *S*_*ij*_. Let *M* be considered mapping data. Then, *M* (*S*_*ij*_) = *N*_*ij*_, where *N*_*i*_ is the evaluation value of the mapping.

The entropy weight ${{\varvec{w}}}_{{\varvec{j}}}$ algorithm involves the following steps:

- *Step 1* Normalize the assessment production base movement index.
  2 $$S_{ij} = \frac{{S_{ij} }}{{\sum\nolimits_{i = 1}^{m} {S_{ij} } }}, \, j = \, 1, \, 2, \cdots ,n$$
- *Step 2* Calculate the relative amount of the production base movement information of the assessment index.
  3 $$S_{j} = - k\sum\nolimits_{i = 1}^{n} {S_{i} (j)\log } \, S_{i} (j).$$
  where the value of *S*_*j*_ is between 0 and 1; and $S_{i} (j) = {{S_{i} (j)} \mathord{\left/ {\vphantom {{S_{i} (j)} {\sum\limits_{i = 1}^{n} {S_{i} (j)} }}} \right. \kern-\nulldelimiterspace} {\sum\limits_{i = 1}^{n} {S_{i} (j)} }},k = {1 \mathord{\left/ {\vphantom {1 {\log n}}} \right. \kern-\nulldelimiterspace} {\log n}}.$
- *Step 3* Calculate the weight of the criterion index.
  4 $$w_{j} = \tfrac{{1 - S_{j} }}{{\sum\nolimits_{j = 1}^{K} {(1 - S_{j} )} }}$$
- *Step 4* Calculate mapping data M.
  5 $$M \, \left( {S_{ij} } \right) \, = w_{j} \times S_{ij}$$

### Definition 3

If mapping data *M* satisfies *M* (*S*_*ij*_) = *N*_*ij*_$\in$* R*, $\in \left[0,1\right]$, *M* can be referred to as the mapping effectiveness measurement. The properties of *M* are as follows:

1. For benefit effectiveness-measuring target of M.
  6 $${\varvec{N}}_{{{\varvec{ij}}}} = \frac{{{\varvec{S}}_{{{\varvec{ij}}}} }}{{\mathop {\max }\nolimits_{{\varvec{i}}} {\varvec{S}}_{{{\varvec{ij}}}} }}.$$
2. For the cost effectiveness-measuring target of M.
  7 $${\varvec{N}}_{{{\varvec{ij}}}} = \frac{{\mathop {\min }\nolimits_{{\varvec{i}}} {\mathbf{S}}_{{{\mathbf{ij}}}} }}{{{\mathbf{S}}_{{{\mathbf{ij}}}} }}.$$
3. For the moderate effectiveness-measuring target of M.
  8 $${\varvec{N}}_{{\varvec{i}}} = \frac{{\mathop {\min }\nolimits_{{\mathbf{i}}} \left\{ {{\varvec{S}}_{{{\varvec{ij}}}} ,{\varvec{S}}_{0} } \right\}}}{{\mathop {\max }\nolimits_{{\mathbf{i}}} \left\{ {{\varvec{S}}_{{{\varvec{ij}}}} ,{\varvec{S}}_{0} } \right\}}},$$
  where ${{\varvec{S}}}_{0}=\frac{1}{{\varvec{n}}}\sum_{{\varvec{i}}=1}^{{\varvec{n}}}{{\varvec{S}}}_{{\varvec{i}}{\varvec{j}}}$ *i* is the index of the measurable production base movement factor, and *j* is the index of the participation in the evaluation needs of decision makers.

### Definition 4

Let the production base movement scenario *E*_*ij*_ have *i* measuring targets. If the mapping of *S*_*ij*_ is *M* (*S*_*ij*_) = *N*_*ij*_, then the synthetic measured effectiveness value, *E*_*ij*_, for one of the events is as follows:

9 $${\varvec{E}}_{{\varvec{i}}}^{\sum } = \mathop \sum \limits_{{{\varvec{j}} = 1}}^{{\varvec{n}}} \frac{1}{{\varvec{n}}}{\varvec{E}}_{{{\varvec{ij}}}} .$$

The associated mapping synthetic effectiveness measuring vectors ${{\varvec{E}}}_{{\varvec{i}}}^{\sum }$ exists and can be expressed as follows:

10 $${\varvec{E}}_{{\varvec{i}}}^{\sum } = \left\{ {{\varvec{E}}_{{{\varvec{i}}1}}^{\sum } ,{\varvec{E}}_{{{\varvec{i}}2}}^{\sum } , \cdots ,{\varvec{E}}_{{{\varvec{ii}}}}^{\sum } } \right\}.$$

### Definition 5

If ${{\varvec{E}}}_{{\varvec{i}}}^{\sum \boldsymbol{*}}$ satisfies the following production base movement condition:

11 $${\mathbf{E}}_{{\mathbf{i}}}^{\sum *} = \mathop {\max }\limits_{{\mathbf{i}}} \left\{ {{\mathbf{E}}_{{{\mathbf{ij}}}}^{\sum } } \right\},{\varvec{i}} \in {\varvec{I}} = \left\{ {1,2, \cdots ,{\varvec{m}}} \right\},$$

then ${{\varvec{E}}}_{{\varvec{i}}{\varvec{j}}}^{\boldsymbol{*}}=\left({{\varvec{x}}}_{{\varvec{i}}},{{\varvec{y}}}_{{\varvec{j}}}^{\boldsymbol{*}}\right)$ are “satisfied production base movement scenarios”; ${{\varvec{y}}}_{{\varvec{j}}}^{\boldsymbol{*}}$ is the decision-maker’s need for a multi-factor screened event, *x*_*i*_, and ${{\varvec{E}}}_{{\varvec{i}}}^{\sum \boldsymbol{*}}$ is the most satisfactory production base movement scenario.

## Case analysis

Founded in 1977, Zong Da Company experienced the most prosperous period in Taiwan's footwear export sales. During this period, the Taiwanese footwear industry began to produce upstream raw materials for other industries, including textile, rubber, and plastics. All materials accounted for 60% of the overall cost of sneakers. Different shoe parts require different materials. Upstream materials had a complete supply chain (e.g., EVA and EVO sole materials from Victory New Materials Limited Company; EVA sponge soles from Asia Plastic Recycle Holding Limited; meshes from Li-Cheng Enterprise Co., Ltd; shoelaces and Velcro from Paiho Group; and synthetic leather from San Fang Chemical Industry Co. Ltd.). In 1991, Zong Da Company moved its production base to China mainly for two reasons: (1) rising wages and a drastic exchange rate increase resulted in a sharp decline in profits, and (2) a high growth rate was estimated in the global sneakers market in the future. However, owing to the 2018 Sino–US trade war, and in the wake of both wage increases as well as the COVID-19 pandemic in 2020, changes occurred in consumption patterns and there were sharp fluctuations in exchange rates, causing the Zong Da Company to actively start considering moving its production base.

**Fig. 2:**
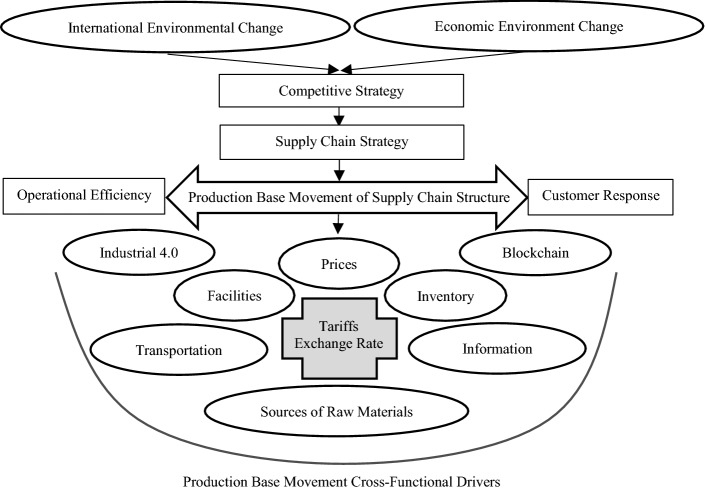
Group knowledge sharing decision-making transfer supply chain model
- *Step 2* Determine the participants. Modgil and Sonwaney (2019) invited 20 experts for consultations regarding the scores for different dimensions, and determined the criteria for the SWARA. In this study, three groups—the footwear industry, government counseling units, and the academic community—participated in the discussion and analysis. A total of 20 participants were included, as shown in Table 2, including six individuals from the footwear industry (finance, procurement, manufacturing, quality control, R&D, and management departments of Zong Da Company), eight individuals from government counseling units (senior officials of the Ministry of Economic Affairs (MEA) and Metal Industries Research & Development Center of MEA), and six individuals from the academic community (professors of sport departments or faculties of colleges, or senior administrators of Taiwan's industry research institutes).

This study used a focus group method (van Bezouw et al. 2019), which allows participants to share their thoughts on a given subject. Interactions and discussions among company members, government officials, and academic experts and scholars were initiated to collect their viewpoints on the production base movement through cross-thinking in a group context, and to share their philosophy, experience, and knowledge of sustainable operation with others. In the context of the current study, GSDEM involved the following steps:

- *Step 1* Determine the research questions and purposes related to supply chain movements in the footwear industry.
  The research questions and purposes of this study were mainly limited to an in-depth investigation into the factors affecting the production base movement in the footwear factory, and the impact of such movement on the supply chain strategy and sustainable development of enterprises. Participating focus groups members could refer to Chopra and Meindl’s (2016) description of the supply chain infrastructure, in which the topics range from a company's competitive strategy, supply chain strategy, industrial 4.0 blockchain production technology, operational efficiency, customer response, and other drivers, to their knowledge management and sharing of inventory, transportation, equipment, information, sources of raw materials, prices. A full discussion of the production line transfer and its impact on the case company is included (Gupta 2020) (see Fig. 2).

**Table 2:** Participants in the focus group

Participating sector	Participating organization	Number of participants	Percentage (%)
Footwear industry	Zong Da company	6	30
Government counseling units	Ministry of Economic Affairs (MEA) and Metal Industries Research and Development Center of MEA	8	40
Academic community	Academic institutions	6	30
- *Step 3* Determine the moderator for discussion of topics regarding the movement of the shoemaking base.
  The moderator, who plays a key role in studies employing this method, is expected to have leadership qualities, a sense of group dynamics, and a deep understanding of the footwear industry. His/her main tasks include discussing the topic of production base movement, selecting the research method for evaluation, controlling the duration of discussion, and determining the location of discussion. Therefore, the moderator of this study was an objective academic representative who had an unbiased position and had participated in many focus group discussions.
- *Step 4* Develop the topics to be discussed.
  The following factors need to be considered for production base movement in the footwear industry: the COVID-19 pandemic, the Sino–US trade war, tariffs, exchange rate, labor costs, government counseling, supply chain, manufacturing technologies, market changes, technological innovation capabilities, and production base movement costs. In addition, the impact of the production base movement on the company’s operations needs to be analyzed. Therefore, the aim of the focus group was to allow participants to share their professional knowledge and experience in the footwear industry, and to discuss the international scenario and market changes so that they can evaluate the feasibility of production base movement with limited resources. The following two topics were proposed:
  - *Topic 1* Determine the criteria for evaluating production base movement, collect information on the internal and external environments of the company as well as the international scenario, and determine the criteria for evaluating the production base movement.
  - *Topic 2* Selecting a research method for evaluating production base movement. After determining the criteria, select an appropriate and objective research method for the evaluation.
- *Step 5* Run the focus group.
*Topic 1: Determining the criteria for evaluating production base movement*
  1. Organisational division
    Zong Da Company has six departments: finance, procurement, manufacturing, quality control, R&D, and management. The finance department is mainly in charge of exchange rate risk management and cost analysis for production base movement. The procurement department is primarily responsible for the assessment of the supply chain. The manufacturing department evaluates the equipment required for production base movement and determines whether to purchase new equipment or replace old equipment. The quality control department evaluates the quality of the supply chain for materials that are moved to other countries. The R&D department is responsible for evaluating the transfer of technical aspects and the transfer of technology for key and innovative products together with the production base. The management department is primarily responsible for assessing the production base movement and providing empirical analysis of language, culture, labor management, education, and training. For data collection, the moderator directly asked questions to the participants and combined the answers with the information collected, including responses pertaining to the exchange rate policy, business tax rate, export tariff, domestic market, and government’s preferential measures in the country to which the production base is to be moved, during the evaluation. Data related to production and supply chains and technologies, which were obtained using the participant observation method, were also used. The moderator encouraged participants to speak actively and share their opinions in order to provide an objective and accurate analysis.
  2. Analysis and discussion
    Zong Da was established in 1977, when US$1 was equivalent to NT$40. In 1991, it moved its production base to China for two reasons: (1) exchange rate fluctuations—$1 was equivalent to NT$26.8 and CNY5.34, respectively (Global Website of the Central Bank of the R.O.C. 2020; Li et al. 2011; investing.com 2020), and (2) increasing wages—the average monthly wage in Taiwan's manufacturing industry was approximately US$676 per month, while that in China’s manufacturing industry was US$78, with the former being 8.67 times greater than the latter (National Bureau of Statistics of the PRC 2020; investing.com 2020) (see Fig. 1). These two factors were unfavorable for the management and development of the footwear industry in Taiwan. Meanwhile, due to a stable exchange rate, low wages, and preferential land and tax policies in China, the company decided to move its production base to China.
  As the COVID-19 pandemic and the Sino–US trade war escalated, US$1 became equivalent to NT$29.27 and CNY 6.79, respectively, in September 2020; the former exchange rate fluctuated by 11.68%, while the latter changed by 27.15% compared with 1991. The average wage in Taiwan was 8.67 times higher than that in China in 1991, although this figure has reduced by a factor of 1.31 times in 2019, see Fig. 1. Exchange rates and wages have significantly reduced companies’ profits. If the production base continues to stay in China, there would be no international competitive advantage; however, this situation would still be favorable for the domestic market. Many shoe manufacturers have directly chosen to shut down factories or have begun to move their production bases to Southeast Asia. The formal launch of the China–ASEAN Free Trade Area has led to competitive threats from countries such as India, Brazil, Vietnam, and Indonesia, where the Red Ocean strategy in terms of costs is applied to low-end shoes. For high-end shoes, the Chinese footwear industry has been following the Blue Ocean Strategy by catering to well-known brands in Italy, Spain, Portugal, and other countries. A comprehensive evaluation of such competitive advantages shows that China's shoemaking costs and export prices have continued to rise, and that the Chinese shoemaking market is gradually losing competitiveness.
  Table 3 lists five emerging production bases, as selected by the representatives of the three focus groups: China, Taiwan, Vietnam, Myanmar, and the Philippines. Relevant data include the average exchange rate changes against the U.S. dollar from October 2016 to September 2019 and the average wage, population, population growth rate, and GDP for 2018. The exchange rate is depreciating only in Taiwan. All the other four countries have witnessed an appreciation in their exchange rates. Regarding wages, only Vietnam and Myanmar have an advantage in terms of shoemaking costs. As for the domestic market, all other countries except Taiwan and Myanmar have a population advantage. However, many shoe factories in China have chosen to shut down or move to other countries due to the effects of payroll and corporate tax rates and stringent environmental policies in China.

**Table 3:** Exchange rate changes, wages, population, and GDP of the five countries (2016–2019) for production base movement Source: National Bureau of Statistics of the PRC (2020); Statistical Information Network of the Republic of China (2020), Taiwan Textile Federation of the R.O.C. (2020), Philippines Daily Minimum Wages (2020), and Aung (2018)

	Average change in exchange rate against U.S. dollar (%)	Average wage in 2019 (USD)	Population in 2020	Population growth from 2016 to 2019 (%)	Average GDP
China	+ 2.1754	1398.77	1.39 billion	0.5	6.7
Taiwan	−1.618	1576.65	23 million	0.115	2.0
Vietnam	+ 4.7	175	95 million	1.1	6.2
Myanmar	+ 21.645	3.55	52.89 million	0.82	6.5
Philippines	+ 11.842	1000	93 million	1.6	6.9
  Based on the results of discussion among the participants, seven evaluation criteria were determined, to decide the countries to which the production base should be moved: labor costs, materials, exchange rate fluctuations, tariffs, supply chain, production base movement costs, and government policies. After the discussion, five alternative solutions for the sustainable operation of the company were preliminarily selected. The advantages, disadvantages, and details of the five solutions are as follows:
  Solution A: Shut down the manufacturing department in China, retain the R&D unit and a production line that can be used for small batch production, and outsource most of the production to other OEMs in China. The reason for this is that in 2016, China’s shoe production reached 13.11 billion pairs, accounting for 57.0% of the world's total shoe production. In 2017, it was 12.62 billion pairs, with a slight reduction compared to 2016. However, China's population advantage, sales channels, and OEM manufacturing industries are all important links in the footwear industry chain. Upstream and downstream manufacturers still have profitability due to their technologies or integration capabilities.
  Advantages: The company only needs to engage in outsourcing management and reduce manufacturing costs.
  Disadvantages: The costs of shoes sold to the U.S. will rise due to uncertainty risks such as tariff and exchange rate changes in the wake of the Sino–US trade war.
  Solution B: Besides Solution A, restart the original production factory in Taiwan, initiate R&D and automatic production of high-priced fitness shoes, and reduce the demand for manual labor. This is because footwear products are daily necessities among consumers. The amount of consumption increases with population growth and disposable income. Increasing awareness regarding health and the importance of sports among people is increasing participation in sports-related activities and driving consumer demand for footwear products. The global fitness shoe market is expected to maintain steady growth. When restarting the production line, the company can introduce Speedfactory, which was established in 2015 by Adidas and uses advanced 3D printing technology, to adopt a production model in which human labor is replaced with robots.
  Advantages: (1) Low-end shoes, using the same production pattern as in Solution A, are produced in China; however, profitability will be worse than before. (2) The production and R&D of high-end shoes are moved to Taiwan. Taiwan has well-educated R&D personnel. Automated production helps increase product value. Initial investments are high, but profitability will also be high in the long run. (3) The Taiwanese government has proposed a southward investment plan, in which the company can establish strategic alliances in Southeast Asian countries to enjoy local government subsidies.
  Disadvantages: (1) Insufficient basic electric power in Taiwan is a concern for the production division. (2) Business tax in Taiwan is slightly higher than that in neighboring countries, but the exchange rate risk is lower in Taiwan.
  Solution C: Move the production base to Vietnam.
  Advantages: Vietnam has a complete supply chain for the footwear industry, which can maintain its existing production and ordering models.
  Disadvantages: Most shoes produced in Vietnam are exported to Europe, and comply with European environmental specifications and requirements. However, the shoes produced by Zong Da Company are exported to the United States rather than Europe, and there will be uncertainties in managing distributors.
  Solution D: Move the production base to Myanmar.
  Advantages: Myanmar has a complete supply chain for the footwear industry, which can maintain its existing production and ordering models.
  Disadvantages: The political environment is unstable, and there are high uncertainty risks associated with exchange rates and domestic tax rates.
  Solution E: Evaluate the export and domestic markets and move the production base to the Philippines.
  Advantages: (1) The economic growth rate of the Philippines is above 6.9%, and consumption is strong. (2) Labor costs in the Philippines are lower than those in other countries. (3) The population is growing rapidly to over 100 million, providing a large domestic market. (4) The government is willing to set up industrial zones that provide tax incentives. (5) Shoes are exported to the U.S. market with a relatively stable exchange rate and tariff preferences.
  Disadvantages: In the Philippines, there is no complete supply chain for the footwear industry, and the wage advantage is not obvious.
- *Step 6* Formulate the criteria and decision-making plans to evaluate the production base movement.
*Topic 2: Selecting a research method for evaluating production base movement*
  The participating scholars proposed a combination of GSDEM and entropy to evaluate solutions. Combining these two research methods has the following advantages: (1) GSDEM is mainly used for dealing with an event and coping with multiple decisions in the event, and can effectively optimize the method of ranking dual-situation solutions. Its advantages include objective evaluation, simple computation, clear principles, and reliable effects. (2) Entropy can be used to calculate the weights of the evaluation criteria based on data randomness. The weights of the evaluation criteria are not affected by the preferences of decision makers, thus achieving objective decision-making for the company’s sustainable operation in the future. The decision-making process for evaluating the production base movement is as follows:
- *Step 6.1*: Calculate the GSDEM data of the evaluation criteria for the five alternative solutions.
  The seven evaluation criteria include: labor costs, materials, exchange rate fluctuations, tariffs, supply chain, transfer costs, and government policies. These criteria were submitted to 20 participants based on historical data and professional judgment. Data (0–1) were analyzed using Eq. (1) and the average GSDEM values of the seven evaluation criteria for the five alternative solutions were obtained, as listed in Table 4.

**Table 4:** Average GSDEM values of participants’ evaluation on production base movement

Alternative	Evaluation Criteria						
	Labor costs	Materials	Exchange rate fluctuations	Tariffs	Supply chain	Transfer costs	Government policies
A	0.60	0.35	0.40	0.75	0.76	0.48	0.73
B	0.35	0.40	0.30	0.69	0.78	0.30	0.34
C	0.40	0.60	0.50	0.62	0.40	0.58	0.83
D	0.35	0.70	0.95	0.68	0.84	0.90	0.90
E	0.45	0.76	0.90	0.45	0.89	0.95	0.80
- *Step 6.2*: Calculate the entropy weights of the seven criteria.
  Equations (2), (3) and (4) were used to calculate the entropy weights of the seven evaluation criteria listed in Table 3, that is, entropy = (labor cost, materials, exchange rate fluctuation, tariff, supply chain, transfer cost, government policy) = (0.1421, 0.1413, 0.151, 0.1360, 0.13917, 0.14875, 0.1415). In this equation, the exchange rate fluctuation is 0.1510, and the transfer cost is 0.14875. The participants opined that these two criteria were the most important for the production base movement.
- S*tep 6.3*: Calculate the GSDEM evaluation data after adjustment.
  Equation (5) was used to obtain the entropy from Step 2 and recalculate the adjusted GSDEM matrix. The calculation results are presented in Table 5.

**Table 5:** Adjusted GSDEM data

Alternative	Evaluation criteria						
	Labor costs	Materials	Exchange rate fluctuations	Tariffs	Supply chain	Transfer costs	Government policies
A	0.09	0.05	0.06	0.10	0.11	0.07	0.10
B	0.05	0.06	0.05	0.09	0.11	0.04	0.05
C	0.06	0.08	0.08	0.08	0.06	0.09	0.12
D	0.05	0.10	0.14	0.09	0.12	0.13	0.13
E	0.06	0.11	0.14	0.06	0.12	0.14	0.11
- *Step 6.4*: Standardize the data.
  Among the seven evaluation criteria, the polarity of the values of two criteria, supply chain and government policies, should be as large as possible, and that of the other five criteria should be as small as possible. Therefore, the data were normalized using Eqs. (6) and (7). The obtained data are listed in Table 6.

**Table 6:** Data standardization and comprehensive GSDEM measure values

Alternative	Evaluation criteria							
	Labor costs	Materials	Exchange rate fluctuations	Tariffs	Supply chain	Transfer costs	Government policies	Grey measure values
A	0.5833	1.0000	0.7500	0.6000	0.8539	0.6250	0.8111	0.7462
B	1.0000	0.8750	1.0000	0.6522	0.8764	1.0000	0.3778	0.8259
C	0.8750	0.5833	0.6000	0.7258	0.4494	0.5172	0.9222	0.6676
D	1.0000	0.5000	0.3158	0.6618	0.9438	0.3333	1.0000	0.6792
E	0.7778	0.4605	0.3333	1.0000	1.0000	0.3158	0.8889	0.6823
- *Step 6.5*: Calculate the comprehensive GSDEM measure data of the solutions.
  The comprehensive GSDEM values were calculated using Eqs. (9), (10) and (11), and the results are listed in the rightmost column of Table 4. The comprehensive GSDEM measure value of Solution B is 0.8259, which is the optimal value among the five alternative solutions. This value is decided by experts and scholars from three parties (footwear industry, government, and academic community). In the future, the company will use Solution B as a strategy for sustainable operations.
  The footwear industry is labor-intensive. It cannot last long in any one place, regardless of location. Almost every 20 years, the shoe industry needs to move to a place where labor is cheaper. All construction begins from scratch. This is also a problem faced by Taiwanese shoe companies, including world-renowned Taiwanese OEMs who have made overseas investments. For example, the Pou Chen Group has factories in Vietnam, Indonesia, Bangladesh, Cambodia, and Myanmar; the Feng Tay Group has production bases in Vietnam, Indonesia, and India; the Ching Luh Group has factories in Vietnam and Indonesia; and the Fulgent Sun Group has investments in Vietnam and Cambodia.
  Therefore, the focus group concluded that Solution B can enable the company to develop sustainably. Low-end shoes should be outsourced to Chinese or other international partner manufacturers. In Taiwan, product upgrades should be conducted, and production bases should not be moved.

## Conclusions

After moving its shoemaking base to China in 1991, Zong Da Company again achieved record-breaking revenue. However, in the wake of the Sino–US trade war, wage increases, and exchange rate fluctuations in 2018, achieving a smooth transition between the old and new business models will be a major challenge for the traditional footwear industry. The conclusions of this study are as follows:

### Implications for theory

Experts from the footwear industry, government, and academic community were invited to jointly construct an objective decision-making model of production base movement for sustainable operations in the footwear industry. The evaluation process is easy and can also solve the problem of production base movement in the footwear industry and enable the sustainable development of enterprises.

In the context of globalization, although the number of manufacturers whose main shoemaking base is located in Taiwan has decreased drastically, the supply chain is not as complete as before 1991. However, Taiwanese manufacturers still have a pivotal position in the global footwear industry. For example, global manufacturers that have established long-term cooperation with Zong Da Company are still the leading footwear manufacturers worldwide and are important partners of international sports brands such as Nike and Adidas.

### Implications for practice

Zong Da Company adopted Solution B as its operational plan. In accordance with this solution, automated production was used suggested to reduce manpower. An operational R&D center should be set up in Taiwan to develop and design new shoe materials and models and to produce high value-added and differentiated products to reduce the risks of the Sino–US trade war and tariff uncertainties. This center should also serve as a raw material management and procurement center that supports production bases in other countries to cope with the dilemma resulting from the reduction of overall scale.

Solutions C, D, and E suggest moving the production base to Southeast Asian countries. With increasing investment in various countries, the entry of industries, and various issues (rising land prices, rapid wage growth, and increasing labor awareness) arising from economic development, the business environment is also deteriorating. Since 2014, issues such as anti-Chinese movements, strikes, and riots have frequently occurred in these countries. Regardless of where the shoemaking base is moved to, the same problems persist in the business environment. Only by carrying out technology R&D, product innovation, and industrial upgrade in Taiwan can enterprises survive sustainably and avoid continuous evaluation of the production base movement issue.

### Limitations and scope for future research

The main limitation of this research is that it analyzes the international situation and economic conditions from the beginning of the Sino–US trade war from 2018 to 2020 before the COVID-19 pandemic. Future research directions include the following:

1. Following the COVID-19, decision B, to outsource the production of low-end shoes to China or other international partners, was optimal, allowing Taiwan to upgrade its products, thereby creating an international division of labor strategy of “receiving orders in Taiwan and producing shoes outside Taiwan.” The company's revenue can reach a new peak.
2. After the COVID-19 pandemic and the Sino-US trade war, at headquarters in Taiwan, the development and upgrade of high-end sports shoes and functional shoes, quotations, material procurement, financial management and control, and so on. The mass production part is outsourced to factories in China or other partner countries for assembly. The verifies the following results: (1) the global sneaker market grew by 9.4%, from US$58.53 billion in 2016 to US$63.223 billion in 2017. The U.S. market grew by 4.1%, from US$23.269 billion to US$22.355 billion (Sporting Goods Intelligence 2020); (2) The growth rate of the sneaker market is also higher than that of the traditional shoe market. Zong Da Company headquarters in Taiwan carry out R&D, 3-D printing, speedfactory, upgrade, quotation, material procurement, and financial management for high-end sneakers (Schwartz 2018).
